# Supplementary material for: Ultrafast transient infrared spectroscopy for probing trapping states in hybrid perovskite films
Source: Commun Chem. 2022 May 30;5:67. doi: 10.1038/s42004-022-00683-7 (PMC9814551; doi:10.1038/s42004-022-00683-7)
Supplement: Supplementary file 1 — Supplementary Information [file 42004_2022_683_MOESM1_ESM.pdf]

Supporting Information

Ultrafast Transient Infrared Spectroscopy for Probing Trapping States in Hybrid Perovskite Films

Ahmed M. El-Zohry[a,b*], Bekir Turedi[c], Abdullah Alsalloum[c], Partha Maity[a], Osman M. Bakr[c], Boon S. Ooi[d], and Omar F. Mohammed[a**]

[a] King Abdullah University of Science and Technology (KAUST), Division of Physical Sciences and Engineering, Thuwal 23955-6900, KSA.
[b] Department of Physics, AlbaNova Center, Stockholm University, 10691 Stockholm, Sweden.
[c] KAUST Catalysis Center, King Abdullah University of Science and Technology (KAUST), Thuwal 23955-6900, Saudi Arabia.
[d] Photonics Laboratory, King Abdullah University of Science and Technology (KAUST), Thuwal 23955-6900, KSA.
* Corresponding author: ahmed.elzohry@fysik.su.se
** Corresponding author: omar.abdelsaboor@kaust.edu.sa

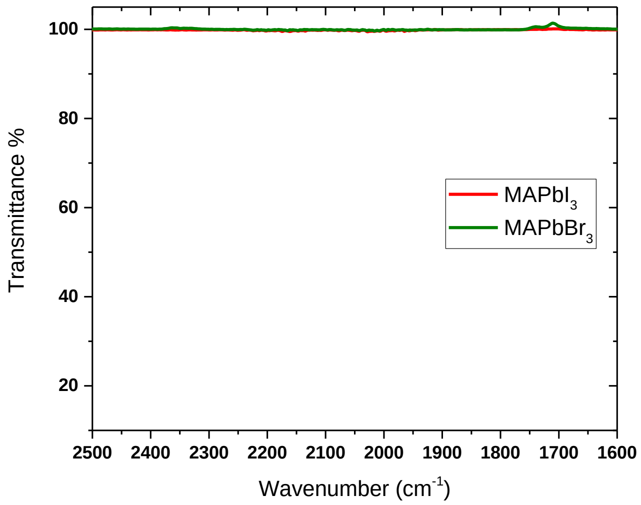

Figure S 1: FTIR for perovskite films in the region of fs-IR measurements.

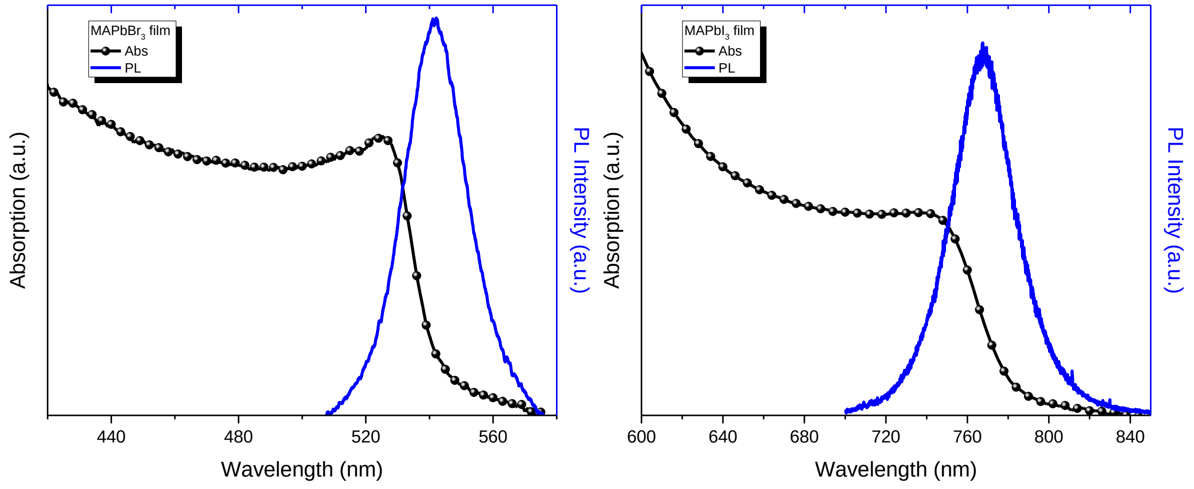

Figure S 2: Steady state normalized absorption and emission data for $MAPbBr_3$ and $MAPbI_3$ films on CaF_2 plates.

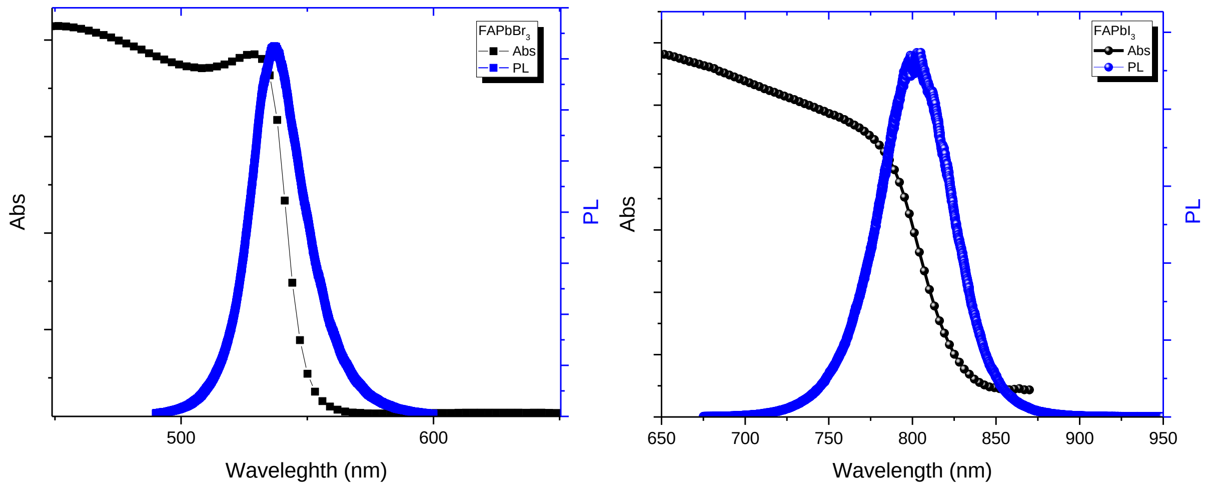

Figure S 3: Steady state normalized absorption and emission data for $FAPbBr_3$ and $FAPbI_3$ films on CaF_2 plates.

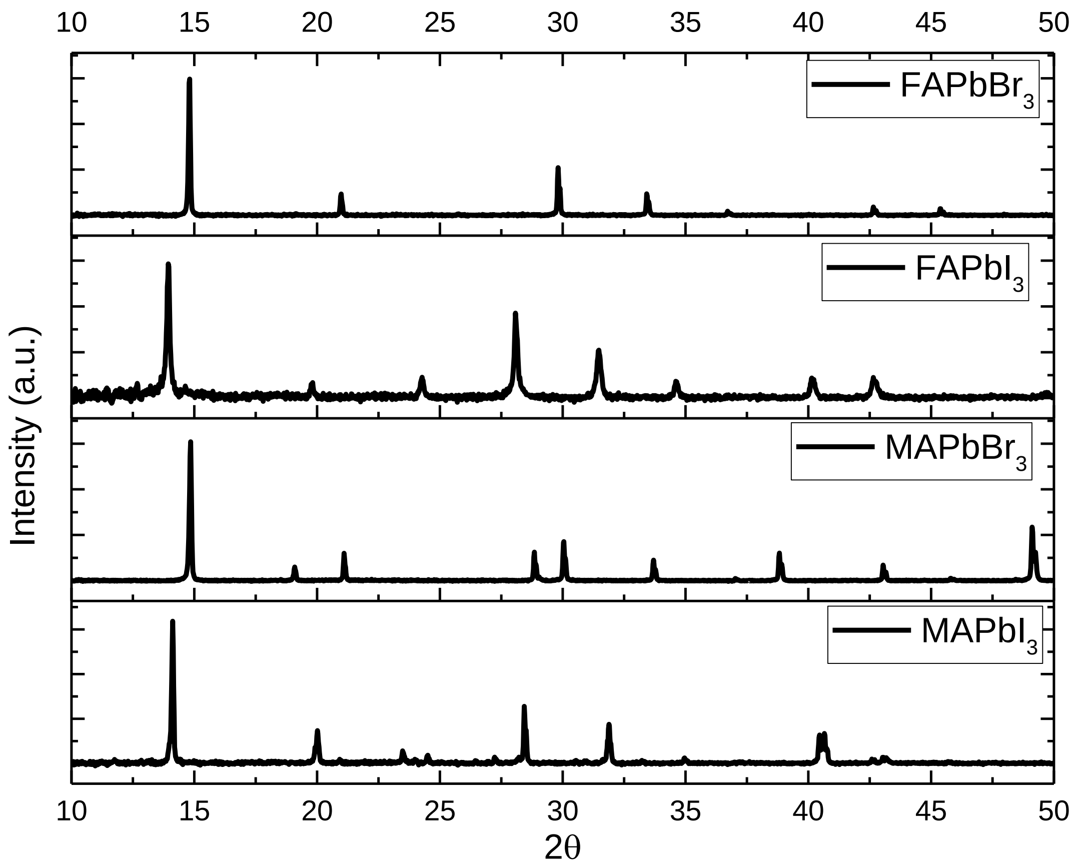

Figure S 4: XRD measurements for thin films of hybrid perovskite films used in the current study.

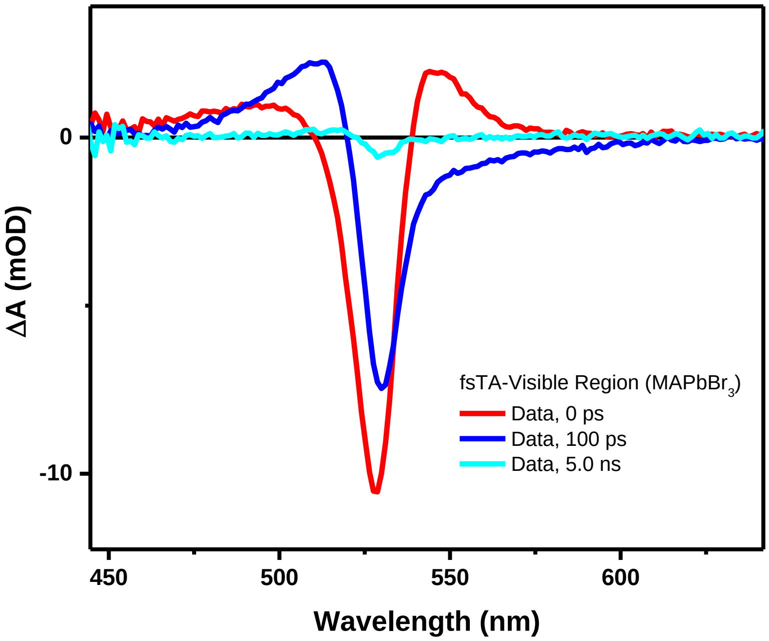

Figure S 5: fs-TA data for $MAPbBr_3$ in the visible region upon exciting with 520 nm.

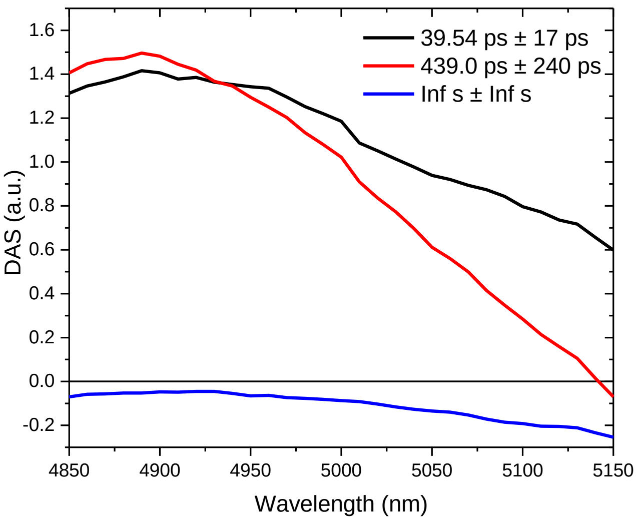

Figure S 6: Decay associated spectra for charge dynamics of $MAPbBr_3$ film

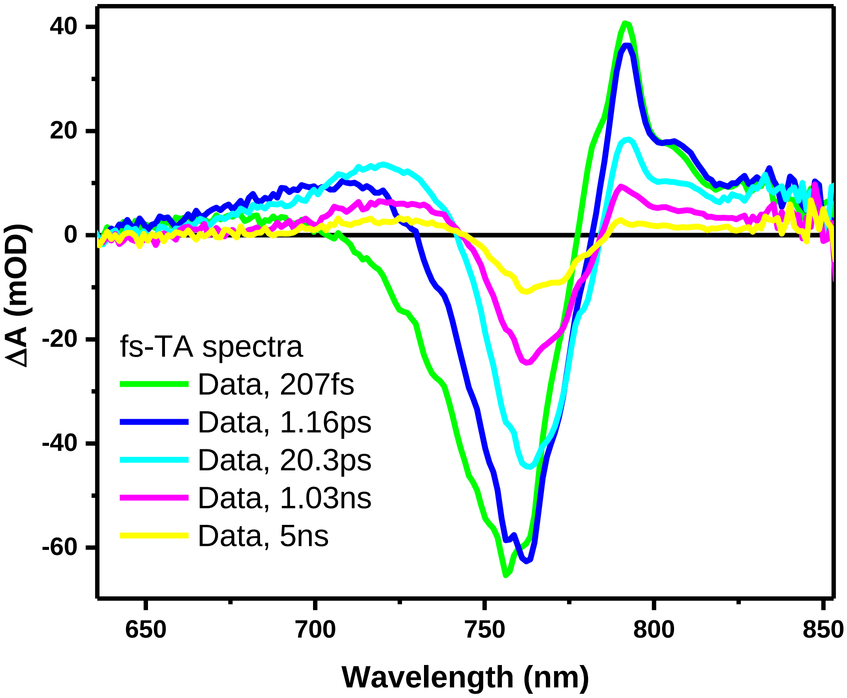

Figure S 7: fs-TA for $MAPbI_3$ in the visible range.

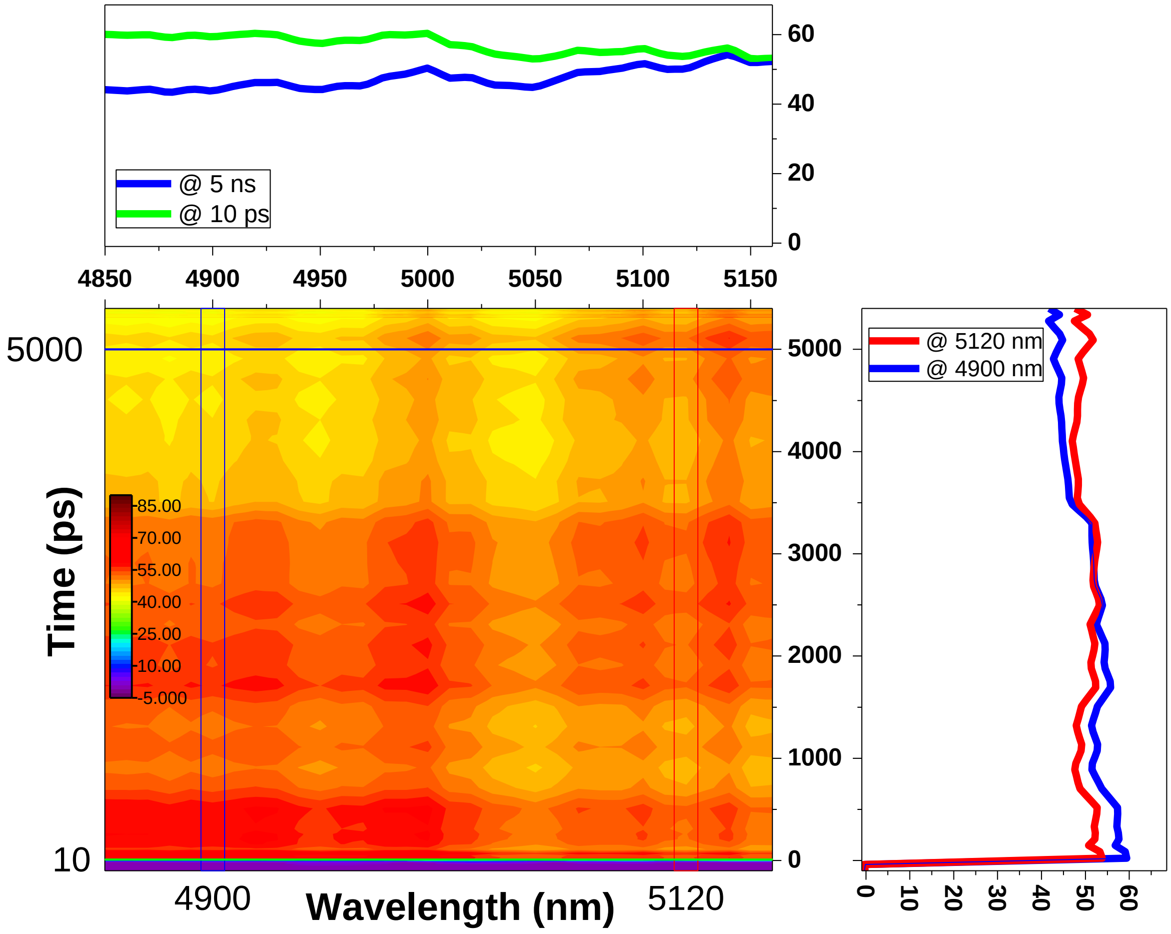

Figure S 8: 2D-false plot of fs-TA for silicon substrate using excitation wavelength of 520 nm and probing the mid-IR window between 4850-5150 nm. Extracted kinetics and spectra are shown at different locations, see the legends.
